# Magnesium Is a Key Regulator of the Balance between Osteoclast and Osteoblast Differentiation in the Presence of Vitamin D_3_

**DOI:** 10.3390/ijms20020385

**Published:** 2019-01-17

**Authors:** Fabiana Mammoli, Sara Castiglioni, Sandra Parenti, Concettina Cappadone, Giovanna Farruggia, Stefano Iotti, Pierpaola Davalli, Jeanette A.M. Maier, Alexis Grande, Chiara Frassineti

**Affiliations:** 1 Department of Life Sciences, University of Modena and Reggio Emilia, 41125 Modena, Italy; Fabiana.mammoli@irst.emr.it; 2 Department of Biomedical and Clinical Sciences “L. Sacco”, University of Milano, 20157 Milano, Italy; sara.castiglioni@unimi.it (S.C); jeanette.maier@unimi.it (J.A.M.M.); 3 Department of Medical and Surgical Sciences, University of Modena and Reggio Emilia, 41125 Modena, Italy; sandra.parenti@unimore.it; 4 Center for Genome Research, University of Modena and Reggio Emilia, 41125 Modena, Italy; 5 Department of Pharmacy and Biotechnology, University of Bologna, 40127 Bologna, Italy; concettina.cappadone@unibo.it (C.C.); giovanna.farruggia@unibo.it (G.F.); stefano.iotti@unibo.it (S.I.); 6 National Institute of Biostructures and Biosystems (NIBB), 00136 Rome, Italy; 7 Department of Biomedical, Metabolic and Neural Sciences, University of Modena and Reggio Emilia, 41125 Modena, Italy; pierpaola.davalli@unimore.it (P.D.); chiara.frassineti@unimore.it (C.F.)

**Keywords:** magnesium, biodegradable magnesium alloys, osteoclasts, hematopoietic U937 cells, human bone-marrow mesenchymal stem cells, vitamin D_3_

## Abstract

Magnesium (Mg) is crucial for bone health. Low concentrations of Mg inhibit the activity of osteoblasts while promoting that of osteoclasts, with the final result of inducing osteopenia. Conversely, little is known about the effects of high concentrations of extracellular Mg on osteoclasts and osteoblasts. Since the differentiation and activation of these cells is coordinated by vitamin D_3_ (VD3), we investigated the effects of high extracellular Mg, as well as its impact on VD3 activity, in these cells. U937 cells were induced to osteoclastic differentiation by VD3 in the presence of supra-physiological concentrations (>1 mM) of extracellular Mg. The effect of high Mg concentrations was also studied in human bone-marrow-derived mesenchymal stem cells (bMSCs) induced to differentiate into osteoblasts by VD3. We demonstrate that high extra-cellular Mg levels potentiate VD3-induced osteoclastic differentiation, while decreasing osteoblastogenesis. We hypothesize that Mg might reprogram VD3 activity on bone remodeling, causing an unbalanced activation of osteoclasts and osteoblasts.

## 1. Introduction

Magnesium (Mg) is the second and fourth most abundant cation in the intracellular compartment and the human body, respectively, where it exists as bound and ionized forms [1,2]. Ionized Mg is the most likely “second messenger”, together with calcium (Ca), for regulating a wide variety of reactions involved in cell response through signal transduction pathways [1,3,4]. Apart from being required for DNA, RNA, and protein synthesis, Mg participates in several biochemical processes acting as a cofactor for hundreds of enzymatic reactions. MgATP^2−^ is both the active Mg species in enzyme binding and the energy-producing form in the cellular active transport [5,6,7,8], although multiple Mg–ATP complexes at different Mg to ATP molar ratio were described ([9] and references cited therein). Also, Mg is the second element in bone after Ca and part of the Mg on bone surface (30%) is exchangeable, acting as a dynamic store to maintain intra- and extracellular Mg levels [10,11]. Imbalances in Mg status such as hypomagnesemia are associated with chronic diseases [12,13,14], while the effect of local high Mg concentrations remains to be clarified [15,16,17]. Mg deficiency is relatively common in the population and may associate with osteoporosis, although it remains to be determined to which extent. Both restricted and increased Mg concentrations affect osteoclast activity in vitro [18,19]. Mg-deficient diets may lead to disorders of bone remodeling, increased osteoclastic activity, and osteoporosis risk in animal models [20,21]. On the other hand, Mg-integrated diets cause Ca deposition in the bone through the interaction with vitamin D_3_ (VD3) [22,23,24,25], thus increasing bone mass in animals or humans to prevent or limit osteoporosis [26,27,28,29]. In recent years, Mg emerged as component of a new class of biodegradable biomaterials for tissue engineering and medical devices to avoid implant removal, as well as to circumvent long-term effects of non-degradable permanent implants. Mg exhibits key advantages especially for load-bearing orthopedic and cardiovascular devices [30,31,32,33,34]. Therefore, mechanisms via which Mg regulates bone repair are under investigation and are fundamental to define the local concentrations of Mg released from the implants [35,36], as well as the effects of local alkalosis accompanying Mg(OH)_2_ dissolution [31]. Biocompatibility of Mg-based implants may be questionable, and effects of Mg alloys degradation on osteogenesis need careful in vitro and in vivo validation studies [30,37,38]. Mg effect on bone progenitor cells is a relevant clinical issue, because the resorption of Mg-based implants in the bone may promote osteoclast differentiation and, consequently, compromise implant efficiency [30]. A gradient of Mg ions from implant might inhibit osteoclastic activity, being later overridden by either receptor activator of nuclear factor κB (RANK)/RANK ligand (RANKL) signaling pathway or inflammatory mediators endowed with osteoclastogenic potential [19,39,40]. Both a reduced [31] and increased number of osteoclasts were detected in the bone surrounding Mg alloy-based implants in animal models [33,41]. Released particulate material and corrosion products from implants seem to attract osteoclasts from neighboring tissue [42]. Alternatively, Mg gradients from implants might hamper VD3 action, which usually coordinates osteoblast and osteoclast balance [43]. Very recently, Mg deficiency was shown to accelerate osteogenic differentiation of human bone-marrow-derived mesenchymal stem cells (bMSCs), partly by generating a stressful condition able to modulate stem cell plasticity and, consequently, cell differentiation potential [44]. 

In the present study, we utilized hematopoietic U937 cells, as a model of osteoclasts derived from hematopoietic precursors, to obtain a homogeneous osteoclastic population devoid of phenotypic and functional differences. The cells were induced to differentiate into osteoclasts by phorbol esters and VD3 [45,46,47,48] in the presence of a range of supra-physiological Mg concentrations (>1 mM). In addition, we analyzed the effect of supra-physiological extracellular Mg on bMSCs induced to osteoblastic differentiation by a cocktail containing VD3 [49]. 

## 2. Results

### 2.1. Analysis of the Effects of High Levels of Mg on the Osteoclastic Differentiation of U937 Cells

**Figure 1:**
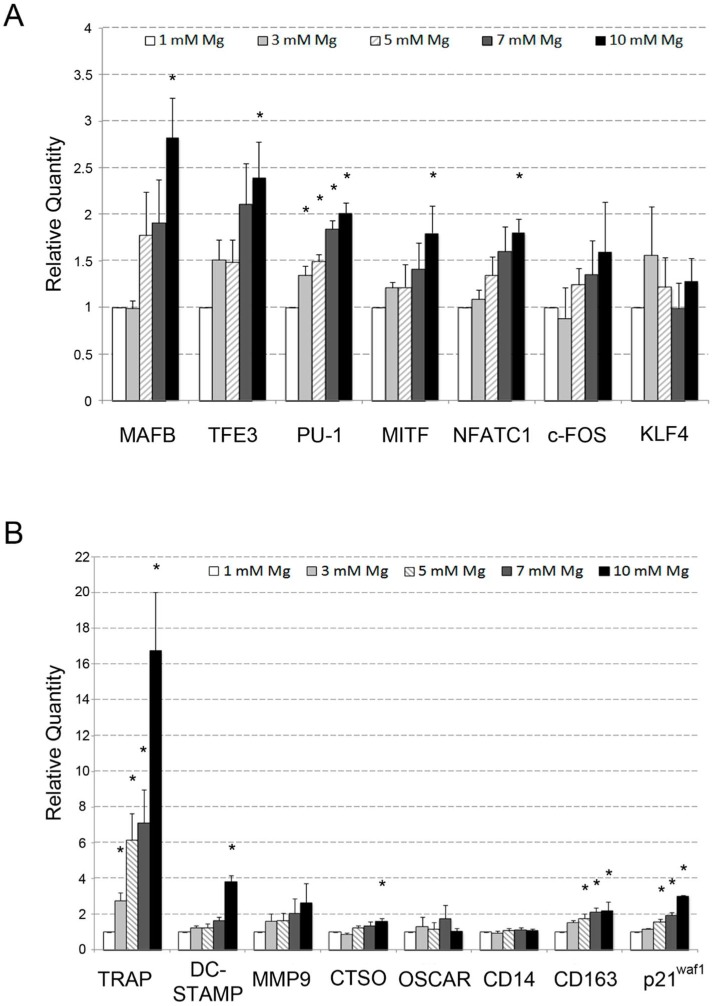
Effects of supra-physiological Mg concentrations on U937 cell-derived osteoclasts. U937 cells were differentiated to osteoclasts upon treatment with phorbol 12-myristate 13-acetate (PMA) and vitamin D_3_ (VD3) for five days and simultaneously exposed to scalar concentrations of Mg ranging from 1 to 10 mM. The messenger RNA (mRNA) expression of transcription factors (**A**) and differentiation markers (**B**), both related to osteoclast differentiation, was then assessed using QRT-PCR. The results obtained are represented as histograms indicating analyzed genes on the *x*-axis and relative quantity of mRNA variations on the *y*-axis. Data are reported as means ± standard error of the mean (SEM) values deriving from a triplicate experiment. Asterisks indicate statistically significant results. *p* < 0.05.

Initially, we assessed whether high levels of extracellular Mg were able to influence osteoclastic differentiation in the presence of VD3. To this purpose, the differentiation of U937 cells to osteoclasts was induced by sequential treatment with phorbol 12-myristate 13-acetate (PMA) and VD3 (see Section 4 for more details). The cells were exposed to Mg concentrations ranging from 1 to 10 mM for the duration of the experiment. It is worth considering that 1 mM is the physiological concentration of Mg, used as a control. At the end of the experiment, the differentiated cells were subjected to (1) QRT-PCR analysis of messenger RNAs (mRNAs) coding for transcription factors and differentiation markers involved in osteoclastic differentiation [18,19,37,50,51,52,53,54]; (2) morphological analysis of cytospins stained with May–Grünwald Giemsa; (3) mono-parametric flow cytometry analysis of cell cycle distribution upon propidium iodide (PI) staining. Using QRT-PCR, we showed that most of the investigated genes underwent an evident upregulation, exhibiting a positive correlation with Mg concentration (Figure 1A). In particular, this expression trend was observed for all the analyzed transcription factors, among which the most upregulated were *MafB* and *Tfe3*, showing a three- and 2.5-fold induction, respectively, followed by *PU.1*, *MITF*, and *NFATC1*, with an approximately twofold increase (upper panel). Similarly, a high concentration of Mg induced the majority of differentiation markers (Figure 1B). In this regard, the most striking were *TRAP* and *DC-STAMP*, both highly specific for osteoclastic differentiation, since they mediate bone demineralization and cell fusion, respectively. The increase in transcript levels, in fact, averaged about 17-fold for the former and fourfold for the latter (lower panel). The *MMP9* and *CTSO* (Cathepsin K) genes, both coding for proteases responsible for degradation of bone extracellular matrix, underwent about twofold increase of mRNA expression.

The non-significant variation of the *OSCAR* gene, coding for an osteoclastic collagen receptor, was not surprising because its induction is strictly dependent on the activation of the RANK/RANKL pathway. This pathway was not planned in our experimental model, in which its biological function was replaced by the similar osteopontin VD3 target gene. As expected, the mRNA levels of the cluster of differentiation 14 (CD14) antigen, typical of the early monocyte phase of osteoclastic differentiation, did not exhibit any variation [55], whereas the late macrophage CD163 antigen exhibited about twofold increase of the same parameter, consistent with the macrophage nature/origin of differentiated osteoclasts [55,56,57]. The approximately threefold induction of p21 growth arrest gene indicated a decrease in proliferation activity, largely expected for a terminal differentiated condition. In general, among all the genes analyzed by QRT- PCR and exhibiting an upregulated mRNA expression in response to high levels of extracellular Mg, five out of seven transcription factors and five out of eight differentiation markers resulted statistically significant at least at the highest (10 mM) Mg concentration. 

**Figure 2:**
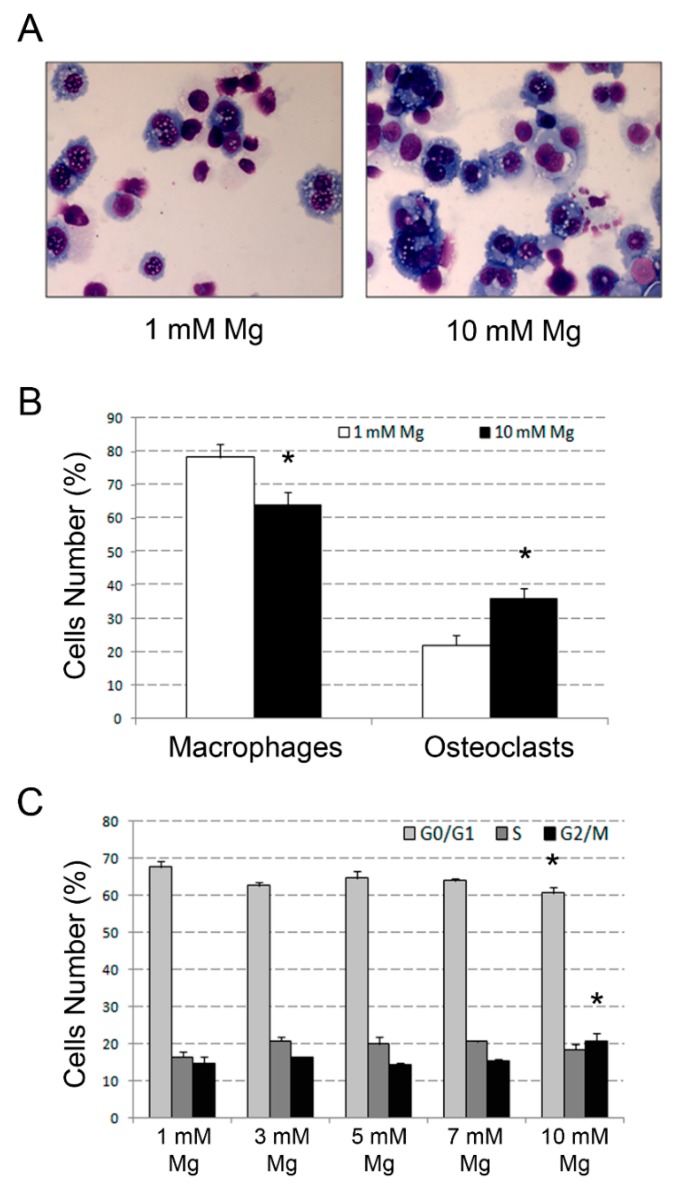
Changes in morphology and proliferation rate in U937 cell-derived osteoclasts after exposure to supra-physiological Mg concentrations. U937 cells, under the experimental conditions described in Figure 1, were subjected to morphological analysis, performed by microscopic examination of May–Grünwald Giemsa-stained cytospins, and cell-cycle assessment, carried out by flow cytometry analysis of propidium iodide (PI)-stained cell suspensions. Panel **A** shows a couple of representative microscopic fields obtained with 1 and 10 mM Mg. The histogram presented in Panel **B** indicate the percentages of macrophages and osteoclasts detected in the same cell samples and relative statistical analysis. Histograms presented in Panel **C** indicate cell-cycle distribution and relative statistical analysis elicited by exposure to scalar concentrations of Mg ranging from 1 to 10 mM. Data are reported as means ± SEM values of a triplicate experiment. Asterisks indicate statistically significant results.

Morphological analysis confirmed QRT-PCR data, showing that exposure to 10 mM Mg determined the increase of mean osteoclast number from 22% to 36% in comparison with 1 mM (*p* < 0.05) (Figure 2A,B). On the same samples, mono-parametric flow cytometry analysis of the cell cycle highlighted a decrease in cell number in the gap (G_0_/G_1_) phases from 68% to 61% (*p* < 0.05), and a concomitant increase of cells in the G_2_/mitosis (M) phases from 15% to 21% (*p* < 0.05), whereas cells in the synthesis (S) phase appeared comparable (16% vs. 18%, respectively; non-significant; Figure 2C).

U937 cells incubated with 1 and 10 mM Mg, in the absence or in the presence of PMA/VD3 were also subjected to quantification of Mg intracellular concentration using a diaza-18-crown-6-hydroxyquinoline (DCHQ5) fluorescent probe. These data showed an increased Mg amount dependent on the Mg extracellular quantity, but more evident in the presence of PMA/VD3 treatment (Table 1). Specifically, 10 mM Mg increased the Mg intracellular amount by about 1.6–1.8 times compared to basal 1 mM both alone (control) and in the presence of PMA + VD3 (treated). Comparing 1 mM Mg alone or with PMA/VD3, the increase was 4.7 times, becoming 5.3 for 10 mM treated versus 10 mM control. Future experiments of treatment with intermediate Mg concentrations will help better characterize this issue.

### 2.2. Comparative Analysis of the Effects Determined by High Level of Mg on Monocyte Differentiation of U937 Cells Induced by VD3 and Macrophage Differentiation of the Same Cells Induced by PMA

**Figure 3:**
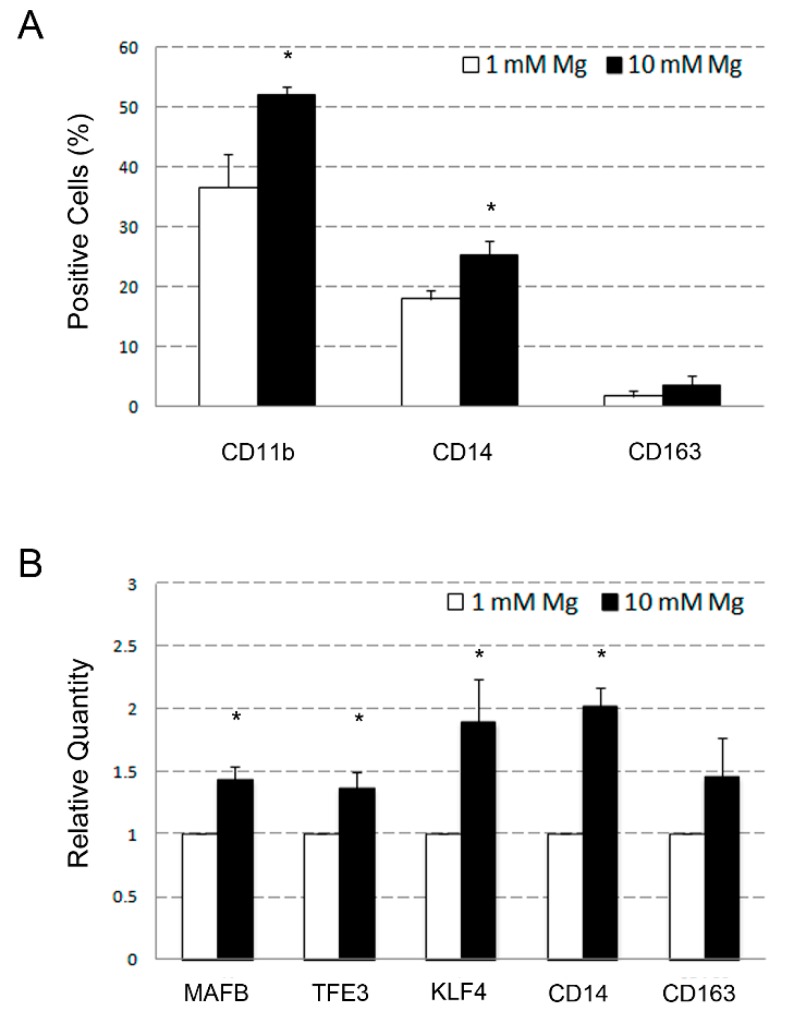
Effects of supra-physiological Mg concentrations determined on U937 cell-derived monocytes. U937 cells were differentiated to monocytes by stimulation with VD3 for five days, and contextually exposed to 1 and 10 mM Mg concentrations. Cell samples were then subjected to flow cytometry and QRT-PCR analysis of typical markers related to the monocyte–macrophage differentiation lineage. Panel **A** shows the results of flow cytometry represented as a histogram, indicating the analyzed surface antigen on the *x*-axis and the percentage of positive cells on the *y*-axis. Panel **B** shows the results of QRT-PCR, indicating analyzed genes on the *x*-axis and the relative quantity of mRNA variations on the *y*-axis. Data are represented as means ± SEM values of a triplicate experiment. Asterisks indicate statistically significant results. *p* < 0.05.

The osteoclastic differentiation of U937 cells results from a combination of effects determined by VD3, an inducer of monocyte differentiation, and PMA, an inducer of macrophage differentiation. Therefore, to better characterize the effect of Mg so far described, we performed an experiment in which U937 cells were separately treated with VD3 to induce monocyte differentiation, and PMA to induce macrophage differentiation. Differentiated cells were then subjected to flow cytometry analysis of CD11b, CD14, and CD163 surface antigens and QRT-PCR analysis of a selected list of genes, i.e., *MAFB, TFE3, KLF4, CD14, CD163*, and *MMP9*. Under these experimental conditions, exposure of VD3-treated U937 cells to 10 mM Mg determined an increase of the mean positivity percentage, compared to 1 mM, from 39% to 53% (*p* < 0.05) for CD11b, 19% to 27% (*p* < 0.05) for CD14, and 2% to 4% (non-significant) for CD163 (Figure 3A). Relative quantities of the analyzed gene transcripts underwent an approximately 1.5-fold increase for *MAFB, TFE3*, and *CD163*, and twofold increase for *KLF4* and *CD14* (all but *CD163* with *p* < 0.05) (Figure 3B). The *MMP9* mRNA resulted undetectable in both the analyzed samples (not shown).

**Figure 4:**
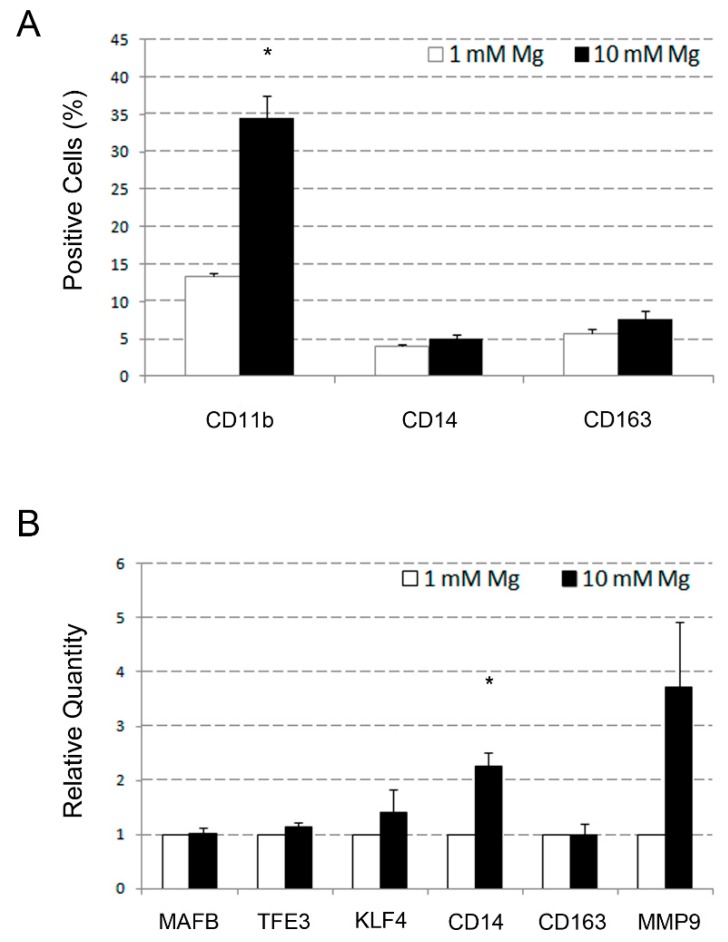
Effects of treatment with supra-physiological Mg concentrations on U937 cell-derived macrophages. U937 cells were differentiated to macrophages by exposure to PMA for two days, and simultaneously exposed to 1 and 10 mM Mg. Cell samples were then analyzed and the results presented as detailed in the legend of Figure 3. (**A**) Results from cell samples subjected to flow cytometry. (**B**) Results from cell samples subjected to QRT-PCR analysis.

Application of the same experimental scheme to PMA-treated U937 cells disclosed an increase of relative quantity of 1.5-fold for *KLF4*, almost 2.5-fold for *CD14*, and fourfold for *MMP9*, but not for *MAFB* and *TFE3*, remaining substantially unvaried (Figure 4B). Among these genes, only the *CD14* exhibited a statistically significant variation, but this result was not confirmed by the flow cytometry analysis of the corresponding protein (4% vs. 5%, non-significant; Figure 4A). The CD163 antigen behaved similarly (6 vs. 8%, non-significant), whereas the CD11b antigen underwent a clear increase from 13% to 33% (*p* < 0.05). Taken together, these data indicate that, although the supra-physiological Mg concentration influenced some effects on PMA-treated U937 cells, its differentiation activity was more evident on VD3-treated U937. This finding suggests that the capacity of Mg to favor the osteoclastic differentiation of U937 cells is most likely VD3-dependent, rather than mediated by PMA. In this regard, a crucial role might be played by the musculoaponeurotic fibrosarcoma oncogene homolog B (MAFB) and transcription factor binding to immunoglobulin heavy constant mu (IGHM) enhancer 3 (TFE3) transcription factors, based on their demonstrated involvement in VD3 response, as well as in osteoclast differentiation [50,51,55,58].

### 2.3. Analysis of the Osteoblastic Differentiation of Human bMSCs in Response to VD3

**Figure 5:**
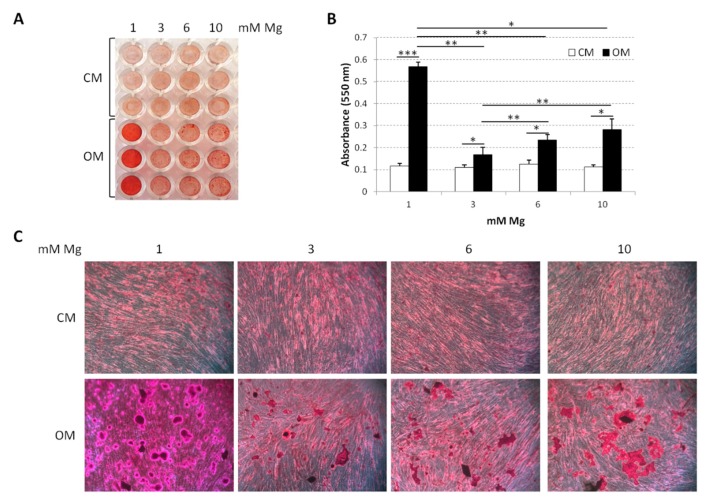
Effects of supra-physiological Mg concentrations on VD3-induced osteoblastic differentiation. Alizarin Red staining was performed on bone-marrow-derived mesenchymal stem cells (bMSCs) cultured in 1, 3, 6, and 10 mM Mg added (OM) or not (CM) with the osteogenic cocktail for 14 days. Whole-well image (**A**) and photographs taken at 10× magnification (**C**) are shown. Absorbance was measured at 550 nm after acid extraction (**B**). * *p* < 0.05, ** *p* < 0.01, *** *p* < 0.001.

After evaluating the osteoclastic differentiation of U937, we focused our attention on osteoblast precursors, i.e., bMSCs. These cells are known to differentiate into osteoblasts, chondrocytes, or adipocytes in response to specific environmental stimuli. In particular, we utilized an osteogenic medium containing VD3, glycerolphosphate, and ascorbic acid (OM) [50,59]. Initially, we evaluated Ca deposition in the extracellular matrix by staining with Alizarin Red S. To this purpose, confluent bMSCs were cultured in medium containing 1, 3, 6, and 10 mM Mg for 14 days either in OM or in their culture medium (CM) as a control. Figure 5 shows a significant reduction of Ca deposits in bMSCs induced to differentiate in medium containing high extracellular Mg (3, 6, and 10 mM) compared to the control cells in physiological Mg concentration (1 mM). We detected a marked reduction of Ca deposition by bMSCs in medium containing 3 mM Mg, while the cells cultured in 6 and 10 mM Mg presented a slight but significant increase of the calcified matrix.

**Figure 6:**
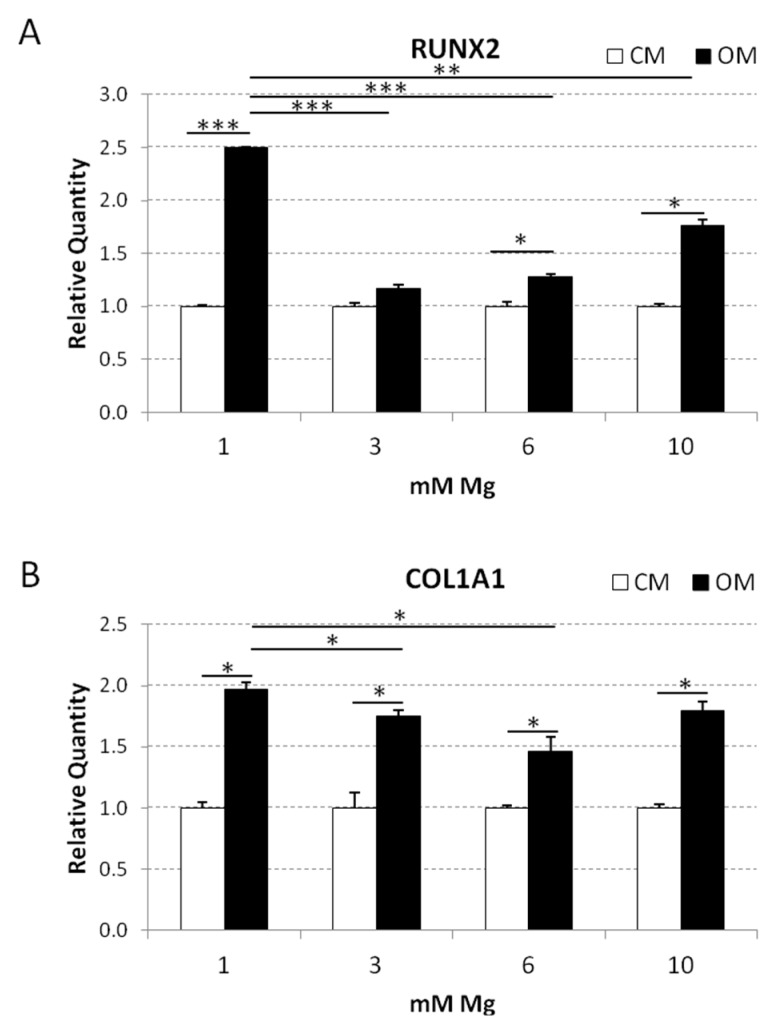
Effects of supra-physiological concentrations of Mg on the expression of *RUNX2* and *COL1A1* in bMSCs exposed to VD3. QRT-PCR was performed on RNA extracted from bMSCs cultured for four days in 1, 3, 6, and 10 mM Mg, added (OM) or not (CM) with the osteogenic cocktail. Primers designed on *RUNX2* and *COL1A1* sequence were used. * *p* < 0.05, ** *p* < 0.01, *** *p* < 0.001.

Then, we analyzed the expression of two osteogenic markers, i.e., *RUNX2*, which is the master switch of osteogenesis, and collagen 1A1 (*COL1A1*), which is essential for the progression of differentiation at early stages. Confluent bMSCs were cultured for four days in CM or OM containing physiological or high Mg. As previously described [59], the osteogenic cocktail induced the expression of *RUNX2* in bMSCs cultured in medium containing 1 mM Mg (2.5-fold increase vs. control in CM) (Figure 6). Interestingly, OM containing 3 mM Mg completely inhibited *RUNX2* expression, while the same medium with 6 and 10 mM Mg slightly induced *RUNX2* expression (1.3- and 1.6-fold increase, respectively). *COL1A1* resulted upregulated in bMSCs induced to differentiate in OM containing 1 mM Mg. In the presence of 3 and 6 mM Mg, *COL1A1* upregulation by the osteogenic cocktail was significantly lower than that in control cells.

**Figure 7:**
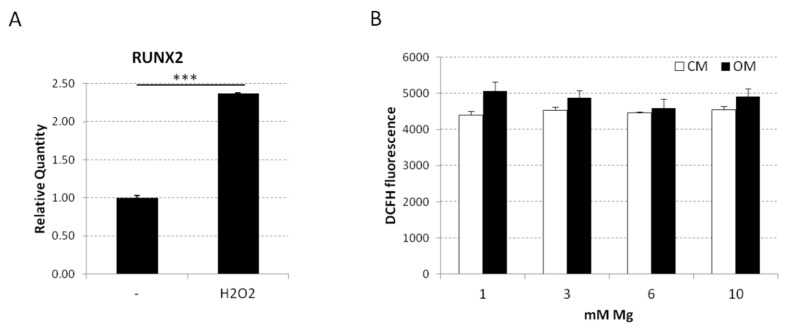
(**A**) The bMSCs were treated for 30 min with H_2_O_2_ (50 μM) and then cultured for four days in culture medium (CM). *RUNX2* expression was analyzed using QRT-PCR. The *p*-value was calculated vs. untreated cells; *** *p* < 0.001. (**B**) Reactive oxygen species (ROS) accumulation was quantified using 2′-7′-dichlorofluorescein diacetate (DCFH, Sigma-Aldrich). Cells were seeded into black-bottomed 96-well plates (Greiner Bio-One) and cultured in 1, 3, 6, and 10 mM Mg, added or not with the osteogenic cocktail for 24 h. Then, cells were washed with phosphate-buffered saline (PBS) and exposed to DCFH (20 µM). The rate of intracellular oxidative stress was evaluated by monitoring the emission at 529 nm of the DCFH dye using a GloMax^®^-Multi Detection System (Promega, Madison, WI, USA). Three independent experiments were performed. Data are shown as means ± standard deviation.

We previously showed that Mg deficiency accelerates bMSC differentiation through a modest increase of the production of reactive oxygen species (ROS) [44]. Accordingly, it is enough to treat the cells with H_2_O_2_ for 30 min and then to keep them in culture medium for four days to enhance *RUNX2* expression (Figure 7A) as detected by QRT-PCR. However, Mg is known to contrast ROS accumulation [60]. To get some insight into the mechanisms involved in the inhibition of bMSC differentiation by high extracellular Mg, we measured ROS production and found no significant differences in ROS generation in cells cultured in high extracellular Mg with or without osteogenic medium (Figure 7B).

## 3. Discussion

The cross-talk between osteoblasts and osteoclasts is regulated by a complex network of juxtacrine, paracrine, and endocrine stimuli. The most important among these signals sustains a positive feedback between osteoblasts and osteoclasts that is aimed at maintaining the so-called bone-remodeling cycle, responsible for the replacement of old bone with new bone [61,62,63,64]. In this context, the di-hydroxylated and active VD3 form represents a typical example of an endocrine signal able to induce a coordinated response of differentiation and activation that involves both osteoblasts and osteoclasts [65,66]. The crucial role played by VD3 in bone metabolism is mainly due to its capacity of upregulating the expression of several bone-related genes acting through a direct transcription mechanism [47]. VD3, in fact, induces osteoblast differentiation, activating the expression of osteocalcin and osteonectin, which, in turn, promote bone formation [67,68]. On the other hand, VD3 also induces osteoclast differentiation, upregulating the expression of osteopontin, which anchors these cells to the bone matrix and allows their activity of bone resorption [69]. 

Magnesium’s role in bone metabolism is widely investigated. Experiments in vitro and in vivo clearly demonstrated that Mg deprivation inhibits osteoblasts and activates osteoclasts, leading to an overall increase of bone resorption [18,24,26,27]. Conversely, only a limited number of reports considered the effects exerted on osteoclasts and osteoblasts by high Mg levels obtaining, in addition, partially controversial results [19,36,37]. Starting from these premises, our work specifically focused on the effects promoted by stimulation with supra-physiological Mg concentrations (>1 mM) in VD3-differentiated osteoclasts and osteoblasts. The aim of this experimental approach was to characterize possible interactions between Mg and VD3 in regulating bone metabolism. The results obtained indicate that high Mg levels potentiate osteoclast differentiation induced by VD3 in U937 cells. This was demonstrated by the upregulated mRNA expression of *TRAP* and *DC-STAMP* genes, which are specific markers of this maturation pathway, and by an increased number of multi-nucleated osteoclasts. The assessment of the transcription factors that could underlie this biological response highlights an upregulation of *MAFB* and *TFE3*, both implicated in osteoclast differentiation [50,51,58]. Although this finding was initially obtained by inducing osteoclast differentiation with a combination of VD3 and PMA, a comparable result was subsequently obtained using VD3 alone. Considering that VD3 is recognized as a monocyte differentiation agent and that monocytes are the upstream precursors of osteoclasts, it is possible to conclude that Mg-promoted effects may begin at early stages of the osteoclast differentiation lineage. Other authors reported that MAFB exerts an inhibitory effect on osteoclast differentiation, but this observation was carried out using RANKL to induce differentiation [69]. Therefore, differences in the pathways investigated and in the experimental conditions could provide a plausible explanation for this data discrepancy. 

As a further result, we found that high Mg levels decrease the osteoblast differentiation of bMSCs induced by an osteogenic cocktail containing VD3. Indeed, a reduction of Ca deposits was particularly evident in bMSCs cultured in 3 mM Mg. Also, RNA levels of *RUNX2* and *COL1A1* were significantly downregulated in response to high Mg exposure, in agreement with the evidence of a lesser matrix calcification in bMSCs. Contrasting results were reported in the literature about the Mg effect on bMSC fate. Some studies on the effect of Mg alloy degradation show cell mineralization induction [70,71], while most of the studies demonstrate that high Mg levels potentiate cell proliferation and inhibit osteogenic differentiation of bMSCs [72,73,74]. It is worthwhile to note that Mg_2_ATP species form at intra-cellular Mg concentration >5 mM to the detriment of MgATP^2−^ [9], the latter being the biological active species required for enzyme activity and, hence, cellular function. Thus, high Mg might impair the activity of one or more Mg-dependent protein kinases, which usually inhibit osteoclast differentiation and promote osteoblast differentiation in physiological conditions. The transcription programs controlling the abovementioned processes could represent the molecular targets of Mg-regulated protein kinases. Altogether, Mg might affect bone remodeling activity of VD3, which commonly coordinates osteoblast and osteoclast activation, giving rise to unbalanced osteoclast activation and consequent bone resorption. Our previous findings showed that low extracellular Mg accelerated the osteogenic differentiation of bMSCs, through an increase of ROS production [44]. Here, we confirm the relevant role of ROS, since exposure for a short time to low concentrations of H_2_O_2_ suffices to upregulate *RUNX2* even in the absence of the osteogenic cocktail. Indeed, ROS are fundamental in promoting cell differentiation partly through autophagy, which is emerging as the most potent accelerator of matrix mineralization and bone homeostasis [75]. Interestingly, we did not observe a modulation of ROS in bMSCs cultured in high-Mg conditions. These results indicate that Mg acts as an anti-oxidant also in bMSCs, and further support the role of ROS in osteogenic differentiation. As demonstrated by Li et al. [76], autophagy inhibition might play a role in the inhibitory effect of high Mg on cell differentiation. Recently, Mg alloys, which show a property profile very close to that of human bone, were used to generate degradable devices for osteosynthesis. While Mg alloy degrades, local Mg concentration in the bone increases. High extracellular Mg is known to inhibit the activity of human primary osteoblasts in vitro [77]. We recall that the Mg role in cell differentiation is rather complex and strictly depends on the cell type [78]. Our findings highlight that high Mg levels can result detrimental for bone not only through inhibition of osteoblast differentiation and function, but also through a parallel increase of osteoclast activity. It has to be pointed out that the present study was entirely carried out using a persistent treatment with supra-physiological Mg concentrations. Therefore, it will be of great interest to conduct a similar study, in the future, by subjecting cell cultures to a transitory stimulation with Mg.

In conclusion, we highlighted translational aspects of potential clinical interest that should be taken into account when utilizing Mg alloy implants. In this regard, it is of crucial importance to better comprehend which are the biological responses of bone upon different Mg-based treatments (diet oral administration or biodegradable surgical implants), which may impact on systemic or local Mg concentration. 

## 4. Materials and Methods

### 4.1. Culture and Differentiation of U937 Cells

The U937 cell line was obtained from the American Type Culture Collection (ATCC; Rockville, MD, USA) and cultured at 37 °C, 5% CO_2_ in Roswell Memorial Park Institute (RPMI-1640) medium (Euroclone, Devon, UK), supplemented with 10% heat-inactivated fetal bovine serum (FBS) (Biowhittaker, Walkersville, MD, USA) and 1 mM l-glutamine (Euroclone). The osteoclast differentiation of U937 cells was induced by a sequential stimulation with 48 nM phorbol 12-myristate 13-acetate (PMA) (Sigma-Aldrich, St. Louis, MO, USA) for two days and then with 10^−8^ M 1α, 25 di-hydroxy vitamin D_3_ (VD3) (Sigma-Aldrich) for a further three days, as already described [45]. The procedure inhibits cell proliferation and generates an osteoclastic population without phenotypic and functional differences [45]. Monocyte and macrophage differentiation of the same cells was induced by exposure to 10^−7^ M VD3 for five days and 48 nM PMA for two days, respectively. [54,56]. Morphological analysis of differentiated U937 cells was performed upon cytocentrifugation, followed by May–Grünwald Giemsa staining.

### 4.2. Culture and Differentiation of bMSCs

Bone mesenchymal stem cells (bMSCs) were isolated from adult human bone marrow recovered from bilateral punctures of the posterior iliac crests of normal volunteers and tested for purity by flow cytometry [44]. The cells were cultured at 37 °C, 5% CO_2_ in Dulbecco’s modified Eagle’s medium (DMEM) added with 1 g/L glucose, 10% heat-inactivated FBS, and 2 mM l-glutamine (all from Sigma-Aldrich) (culture medium, CM). Osteogenic differentiation was induced once the cells reached confluence using bMSCs between passage 2 and 5, and exposing them to an osteogenic cocktail containing 2 × 10^−8^ M VD3, 10 mM β-glycerolphosphate, and 0.05 mM ascorbic acid (all from Sigma-Aldrich) (osteogenic medium, OM). Ca deposition by bMSCs was evaluated on cells rinsed with phosphate-buffered saline (PBS), fixed with 70% ethanol for 1 h, and stained for 10 min with 2% Alizarin Red S (pH 4.2) [59]. Then, Alizarin Red S staining was released from the cell matrix by incubation for 15 min with 10% cetylpyridinium chloride dissolved in 10 mM sodium phosphate (pH 7.0) (all from Sigma-Aldrich); finally, the absorbance was measured at 562 nm. 

### 4.3. Reactive Oxygen Species Evaluation

Intracellular oxidative stress was quantified using 2′-7′-dichlorofluorescein diacetate (DCFH, Sigma-Aldrich, cat. no. 35845). Cells were seeded into black-bottomed 96-well plates (Greiner Bio-One, Frickenhausen, Germany), cultured in medium containing 1, 3, 6, or 10 mM Mg. The cells were then washed with PBS and exposed to DCFH (20 μM). The rate of intracellular oxidative stress was evaluated by monitoring the emission at 529 nm of the DCFH dye using a GloMax^®^-Multi Detection System (Promega, Madison, WI, USA). Three independent experiments were performed. Data were shown as means ± standard deviation.

### 4.4. Flow Cytometry Analysis

Cell-cycle distribution was assessed by mono-parametric flow cytometry analysis of U937 cells upon a 30-min incubation at 4 °C with an hypotonic fluorochrome solution containing 50 μg/mL propidium iodide (PI), 0.1% sodium citrate, and 0.1% Triton X-100 (all from Sigma-Aldrich). Evaluation of surface differentiation antigens was carried out using the following monoclonal antibodies (MoAb): phycoerythrin-conjugated (PE) mouse anti-human CD11b MoAb, fluorescein isothiocyanate-conjugated mouse anti-human CD14 MoAb, and PE-conjugated mouse anti-human CD163, all from Miltenyi Biotec, Auburn, CA. Negative controls were performed using isotype-matched nonspecific antibodies (Miltenyi). Each antibody was incubated, at the proper dilution, with cell samples, in PBS containing 5% fetal calf serum (FCS) and 1% FcR blocking reagent (Miltenyi), for 30 min at 4 °C. Cells were then washed twice and re-suspended with PBS. All samples undergoing flow cytometry were finally analyzed using a Coulter Epics XL flow cytometer (Coulter Electronics Inc., Hialeah, FL) [79]. 

### 4.5. RNA Extraction and QRT-PCR Reaction

Total RNA was isolated using the Qiagen total RNA purification kit (Qiagen, Valencia, CA, USA) and, once extracted, its integrity and concentration were assessed using a NanoDrop 2000 spectrophotometer (Thermo Fisher Scientific, Waltham, MA, USA). Quantitative real-time PCR (QRT-PCR) was performed with an ABI PRISM 7900 sequence detection system (Applied Biosystems, Foster City, CA, USA) on 100 ng of total RNA reverse-transcribed using the High-Capacity complementary DNA (cDNA) Archive Kit (Applied Biosystems). Each cDNA sample was run in triplicate using primers and probes supplied by Applied Biosystems as pre-made solutions and the Faststart Universal Probe Master Mix (Roche Diagnostics, Mannheim, Germany) containing all the reagents necessary for amplification. Normalization of signals was obtained using the glyceraldehyde 3-phosphate dehydrogenase (GAPDH) mRNA as an endogenous control. Statistical analysis of QRT-PCR results was conducted using the (2^−∆∆*C*t^) method, which calculates relative changes in gene expression of the considered target mRNA normalized to the endogenous control and related to a calibrator sample. The values obtained were represented in terms of relative quantity of mRNA level variations [80,81].

### 4.6. Quantification of Total Cell Mg by Spectrofluorimetric Assay

Intracellular total Mg content was accurately quantified on sonicated samples of U937 cells by employing the fluorescent dye DCHQ5, as previously described in detail [82,83]. We developed a chemical synthesis of this dye in order to obtain an Mg determination on a very small number of cells and to map intracellular Mg distribution and movements.

### 4.7. Statistical Analysis

All experiments were repeated at least three times, and results were presented as means ± standard error of the mean (SEM) values. Pairwise comparisons were carried out using a Student’s *t*-test procedure. Results of statistical analysis were considered significant when exhibiting *p*-values ≤0.05, as indicated by asterisks.

## 5. Conclusions

The present results offer new insights into Mg and VD3 interactions in coordinating osteoblast and osteoclast differentiation and activation, and in the subsequent bone remodeling.

We demonstrated here that supra-physiological levels of intracellular Mg cause opposite effects in osteoclast and osteoblast differentiation, as they potentiate VD3-induced osteoclast differentiation in U937 cells, while they inhibit VD3-induced osteoblast differentiation in bMSCs. These observations prompt an investigation of Mg effect in reprogramming VD3 action on bone remodeling in both physiological and pathological conditions.

## Figures and Tables

**Table 1:** Effects of phorbol 12-myristate 13-acetate (PMA)/vitamin D_3_ (VD3) on the total Mg intracellular concentration. Measurements were carried out in U937 cells incubated with 1 and 10 mM Mg, in the absence (control) or presence of PMA/VD3 (treated). Cells were sonicated and the Mg concentration determined using the fluorescent probe diaza-18-crown-6-hydroxyquinoline (DCHQ5). Data are reported as means ± standard error of the mean (SEM) values of a triplicate experiment.

Mg Extracellular Concentration	Mg Intracellular Concentration (nmol/10^6^ cells) Mean ± SEM
Control (1 mM)	12.2 ± 0.0
Control (10 mM)	19.1 ± 2.1
PMA + VD3 (1 mM)	57.2 ± 2.0
PMA + VD3 (10 mM)	101.6 ± 13.5

## Abbreviations

bMSCs	Bone-marrow mesenchymal stem cells
COL1A1	Collagen type I alpha 1 chain
CTSO	Cathepsin o gene
DCFH	20-70-Dichlorofluorescein diacetate
DC-STAMP	Dendritic cell-specific transmembrane protein
KLF4	Kruppel-like factor 4
MafB	Transcription factor MafB
MMP9	Matrix metallopeptidase 9
MITF	Microphthalmia-associated transcription factor
NFATC1	Nuclear factor of activated T cells c1
PMA	Phorbol 12-myristate 13-acetate
PU.1	PU.1 transcription factor
ROS	Reactive oxygen species
RUNX2	Runt-related transcription factor 2
Tfe3	Transcription factor E3
TRAP	Tartrate-resistant acid phosphatase
VD3	25 di-hydroxy vitamin D_3_
